# Green synthesis of PEGylated iron oxide nanoparticles of *Eriobotrya japonica* leaves extract in combination with B3, against *Plasmodium falciparum* 3D7 strain

**DOI:** 10.1186/s41182-025-00733-5

**Published:** 2025-04-28

**Authors:** Soudabeh Etemadi, Ahmad Mehravaran, Edris Yousefi Delcheh, Aram Khezri, Mehdi Nateghpour, Afsaneh Motevalli Haghi, Ahmad Gholami

**Affiliations:** 1 Department of Medical Parasitology and Mycology, School of Medicine, Zahedan University of Medical Sciences, Zahedan, Iran; 2 Department of Medical Laboratory Science, Sirjan School of Medical Sciences, Sirjan, Iran; 3 Infectious Diseases and Tropical Medicine Research Center, Research Institute of Cellular and Molecular Science in Infectious Diseases, Zahedan University of Medical Sciences, Zahedan, Iran; 4 Department of Medical Parasitology and Mycology, School of Public Health, Tehran University of Medical Sciences, Tehran, Iran; 5 Department of Pharmaceutical Nanotechnology, Faculty of Pharmacy, Tehran University of Medicine Sciences, Tehran, Iran

**Keywords:** Green synthesis, Polyethylene glycol, Iron oxide nanoparticles, Eriobotrya japonica, Plasmodium falciparum

## Abstract

**Background:**

*Plasmodium falciparum* represents the most prevalent and lethal protozoan responsible for malaria in humans. This investigation aims to synthesize iron nanoparticles utilizing the polyethylene glycol (PEG) synthesis approach with an *Eriobotrya japonica* leaves extract and investigating its anti- *P. falciparum* activity in the in vitro environment in combination with nicotinamide and comparing its effect with chloroquine.

**Methods:**

Iron oxide nanoparticles were synthesized using *Eriobotrya japonica* leaf extract through a green synthesis method. The physicochemical properties of the nanoparticles were analyzed using DLS, FESEM, FTIR, XRD, and MTT assays. During the initial phase, varying concentrations of Japanese parsnip leaf extract, nicotinamide, iron nanoparticles synthesized through the PEGylated green synthesis technique, and chloroquine (as a control pharmacological agent) were individually administered to the culture medium of *P. falciparum* 3D7. Subsequently, the synergistic IC50 effects of these compounds were evaluated in relation to one another using the FIX RATIO methodology applied to the culture medium.

**Results:**

The DLS evaluation of iron oxide nanoparticles showed an average hydrodynamic size of 155 nm. The XRD examination exhibited the crystallinity of the particles. SEM images recognized the spherical nature of synthesized Fe_3_O_4_ nanoparticles. The relative combination of plant extract–nicotinamide had a synergistic effect and the best dose was observed in 70% plant extract–30% nicotinamide, resulting in a 70% reduction in parasitic load. The most pronounced growth-inhibitory effect was observed in the formulation comprising 50% PEGylated green synthesized Fe_3_O_4_ nanoparticles and 50% nicotinamide, yielding a 73% inhibition rate.

**Conclusions:**

The presence of a synergistic effect was evident across all combinations of plant extract–nicotinamide and iron oxide nanoparticles synthesized through the PEGylated green synthesis approach. Furthermore, the methodologies of green synthesis and PEGylation of iron oxide nanoparticles are deemed effective strategies for enhancing stability, minimizing toxicity, reducing particle size, and facilitating improved precision and efficacy in the application of these entities within biomedical research contexts.

## Introduction

Malaria is an infectious disease that threatens human life and is one of the main causes of death in tropical and subtropical countries. According to the latest report by the WHO, in 2022, there will be 247 million new cases of malaria and 619 thousand deaths due to this disease [1]. Among the myriad of clinical manifestations associated with malaria in humans, include elevated fever, weakness, nausea, and headache, and in acute cases, the disease causes jaundice, epilepsy, coma, and death [2]. *Plasmodium falciparum*, widely recognized as the most prevalent and lethal protozoan parasite species, is a significant agent of morbidity and mortality in this context [3]. The only strain that exists in the Iranian Microorganism Bank and can be cultivated is the *Plasmodium falciparum* 3D7 strain. Due to the successive passages in the bank, this strain is only available at Tehran University of Medical Sciences. Due to the considerable documentation of parasite resistance to pharmacological agents such as chloroquine, amodiaquine, sulfadoxine, quinine, mefloquine, and artemisinin in recent years [4–8], associated with the multitude of adverse effects with these medications, vital for investigating a novel compound derived from botanical extracts to combat *Plasmodium falciparum* is undeniable [9–11].

*E. japonica* is an evergreen tree in subtropical regions, cultivated for over 2,000 years. This plant's leaves and fruits contain numerous beneficial compounds and have been traditionally used for various therapeutic purposes, including treating diabetes, aiding in kidney stone removal, and addressing liver issues [12]. The leaves of Japanese parsley are rich in antioxidants, anti-inflammatory compounds, fatty acids, phenolics, and terpenoids, many of which have demonstrated anti-parasitic properties [13]. The terpenoids found in the Japanese parsley leaves, such as ursolic acid, interfere with the reproduction of *Plasmodium falciparum* by targeting the enzyme PfHGXPT. Additionally, maslinic acid has been shown to inhibit the schizont stage of the parasite [14]. The polyphenols of the plant leaves include caffeic acid, gallic acid and catechin, all of which have been proven to have inhibitory effects on *Plasmodium falciparum* [15]. The leaves of Japanese parsley contain various compounds, including polyphenols and fatty acids such as linoleic acid, linolenic acid, oleic acid, and gallic acid. Each of these compounds has demonstrated significant inhibitory effects on the growth of *Plasmodium falciparum* [15].

In addition, over the past decade, the use of iron nanoparticles (IONPs) has become increasingly popular in applications such as immunoassays, cell tracking, tissue repair, and drug delivery. This rise in usage is attributed to their unique physicochemical characteristics, low toxicity, chemical stability, and beneficial biological properties [16]. Iron oxide nanoparticles (Fe_3_O_4_) exhibit anti-Plasmodium activity on their own and have been used in combination with other drugs to combat drug resistance [17, 18]. Green chemistry is a fascinating field of current research in physical and biochemical sciences [19]. Synthesis of functional nanomaterials is flourishing as the most favorable domain of researchers due to their immense applications like in bimolecular imaging, therapeutics, drugs delivery, biomedicines, cancer treatment, cosmetic surgery and molecular-based detection, etc. [20]. Traditional physical and chemical methods for producing nanoparticles are often expensive, harmful to the environment, and require specialized facilities. Additionally, these methods typically involve the use of toxic agents, some of which may be carcinogenic, making them unsuitable for biological applications. In recent years, green synthesis methods for nanoparticles have gained attention for being effective, environmentally friendly, non-toxic, and inexpensive [21]. Microorganisms and plant extracts can be used in green synthesis process. Research shows that plant extracts significantly increase the rate of reduction of metal ions and their stability compared to microorganisms [21, 22].

In the process of green synthesis, plant extracts convert iron into non-toxic nanoparticles. This biological activity allows iron nanoparticles to act as drug carriers in later stages of research, helping to reduce drug resistance, increase local concentration, and act as passive cellular and tissue targeting agents to improve selectivity [23]. Polyethylene glycol is utilized in medical nanoparticle research for drug delivery and surface coating, enhancing stability while reducing the toxicity of nanoparticles [24]. The use of polyethylene glycol in synthesizing iron oxide nanoparticles modifies their surface charge, stability, size reduction, and toxicity. This study employed polyethylene glycol for this specific purpose [25]. Additionally, nicotinamide (known as Vitamin B3) is a water-soluble amide derivative that multiple studies have demonstrated can enhance the effectiveness of anti-*Plasmodium falciparum* medications [26]. This vitamin is capable of inhibiting the activity of histone deacetylase in the Sirtuin transcription factor (Sir2), thereby inhibiting the growth of *Plasmodium falciparum* [27]. Combination therapy has always been a consideration for the World Health Organization (WHO) due to its potential to delay the development of resistance in parasites and generate positive results. The aim of this project is to utilize iron nitrate particles combined with the aqueous extract of Japanese parsnip leaves (*Eriobotrya japonica*) to produce iron nanoparticles through a green PEGylated synthesis method. Additionally, we will investigate the anti-*Plasmodium falciparum* activity of these nanoparticles in an in vitro environment, in combination with nicotinamide, and compare their effectiveness to that of chloroquine.

## Materials and methods

### Extraction from the plant

The leaves of the Japanese parsnip plant were collected, cleaned, sun-dried, and then ground into a powder. To prepare the hydroalcoholic extract, 100 g of the powdered plant leaves were mixed with a solution of 20% water and 80% ethanol (96%) to a total volume of 600 ml. This mixture was rotated at 325 rpm for 24 h. A sterile cloth was used to remove coarse sediments, and the liquid extract was filtered through the Whatman No. 4 filter paper. The liquid extract solvent was then evaporated using a laboratory oven at 40 degrees Celsius for 4 h, resulting in the final precipitated extract.

### Preparation of iron oxide nanoparticles by green PEGylated synthesis method

Iron nitrate nonahydrate (99.99%) and iron sulfate heptahydrate (99.5%) were purchased from Ghatran Chimi Company, Iran. Nicotinamide laboratory powder was sourced from Sigma Aldrich Company, while chloroquine diphosphate laboratory powder was obtained from UDP Company. Polyethylene glycol 600 (PEG-600) liquid was purchased from Merck. For the green synthesis of iron oxide nanoparticle (Fe_3_O_4_), a mixture of 9% iron nitrate and 7% iron sulfate was added to a liquid form of plant extract (before evaporation and the use of a laboratory oven). This mixture was combined with polyethylene glycol, which was dissolved at a temperature of 60 degrees Celsius for 30 min while being stirred on a heater. Following this, 30% hydrochloric acid (HCl) and 25% ammonium were added to adjust the pH to above 11. After stirring for 5 min, a dark brown precipitate appeared in the solution. In this process, the liquid plant extract serves both as a solvent and as a reducing agent, facilitating the production of nanoparticles from metal salts. The nanoparticles obtained were dried using a laboratory oven.

### Exploring the properties of iron oxide nanoparticles

#### Dynamic light scattering (DLS)

Dynamic light scattering (DLS) is a technique used to measure the size and electrical charge of particles in the nanometer to micron range. In this method, the light scattered by a suspension exhibits oscillations, which are recorded by placing a detector at a specific angle to capture the intensity of the scattered light over a set period. DLS provides a quick way to determine the diameter of particles. This technique was employed to measure the size, dispersion range, and electric charge of iron oxide nanoparticles (Fe_3_O_4_) produced through both chemical methods and green PEGylated synthesis.

### Field emission scanning electron microscope (FESEM)

A field emission scanning electron microscope (KYKY EM8000 F FESEM-China) was used to observe the surface morphology, composition of chemical elements, and consistency of iron oxide nanoparticles obtained by the green dye synthesis method. For this purpose, some of the diluted powder suspension was prepared on the glass, and a thin gold coating was placed on its surface in vacuum before microscopic scanning and observed at 25 kV in vacuum. X-ray energy diffraction spectroscopy (EDS) was also performed to detect the percentage of elements in solid powder samples.

### Fourier transform infrared spectroscopy (FTIR)

FTIR method was used to identify bonds and functional groups present in iron oxide nanoparticles (Fe_3_O_4_) obtained by chemical method and PEGylated green synthesis and to check the amount of loading of the extract in the nanoparticle composition. For this analysis, the nanoparticle powder obtained by the chemical method and the PEGylated green synthesis was placed on the ATR crystal in the size of a thin layer of the sample (about 2 mm) and pressed using a rotary press, to ensure optimal contact between the sample and the crystal.

### Hemolytic test

To conduct the hemolytic test on the hydroalcoholic extract of Japanese parsnip, nicotinamide, and green-synthesized nanoparticles, 5 mL of fresh whole blood with heparin anticoagulant was obtained from a healthy volunteer. After centrifugation and washing with PBS, 1% blood suspension was prepared. In each well of three microplates, 100 µL of the prepared blood was added. Subsequently, 100 µL of the hydroalcoholic extract, nicotinamide, and green-synthesized nanoparticles with concentrations of 0.1, 1, 10, 50, 100 and 200 µg/mL, were added into the second to seventh wells of all three microplates, respectively. The first wells served as negative controls, containing an equal volume of PBS, while the eighth wells were positive controls, containing 100 µL of Triton X-100. After 3 h of incubation and centrifugation, the optical absorbance of the supernatant was measured at a wavelength of 540 nm. The percentage of drug-induced hemolysis was calculated using the following formula [28]:

$${\mathbf{Percenthemolysis}}\% = \frac{{{\mathbf{OD}}\,\,{\mathbf{test}} - {\mathbf{OD}}\,\,{\mathbf{negative}}\,{\mathbf{control}}}}{{{\mathbf{OD}}\,{\mathbf{positive}}\,{\mathbf{control}} - {\mathbf{OD}}\,{\mathbf{negative}}\,{\mathbf{control}}}} \times 100.$$

Statistical analysis was done using GraphPad prism9 software and the results were analyzed with one-way ANOVA. The significance level of the differences was P < 0.05.

### Cytotoxicity test (MTT)

To perform this test, a breast cancer epithelial cell line (MCF7) was used. This cell line was obtained from Zahedan Infectious and Tropical Diseases Research Center. Investigating the cytotoxic effect of hydroalcoholic extract of Japanese parsnip leaves, nicotinamide, and PEGylated nanoparticle, green synthesized by colorimetric method, using tetrazolium 3-(4,5-dimethylthiazol-2yl)−2,5-diphenyl bromide) was performed. This method is based on the activity of the mitochondrial succinate dehydrogenase enzyme of living cells, which turns the yellow MTT solution into purple formazan crystals, which after dissolving in DMSO can be measured in the ELISA rider device. 180 µl of cell suspension were poured into each well of a 96-well plate in such a way that each milliliter of culture medium had 3 × 10^4^ cells, then 20 µL of different concentrations were added. The drugs were introduced into the plate's wells. Doxorubicin was used as a positive control and culture medium containing 0.5% DMSO without any drug was considered as a negative control. After 48 h of incubation, 20 µL of MTT solution (5 mg/mL) was added to each well. After 2 h of incubation, 100 µL of DMSO was replaced with the previous solution to dissolve the formazan crystals, and the optical absorption of all three plates was read at a wavelength of 560 nm by an ELISA rider device. 3 repetitions were determined for different dilutions of each substance. The percentage of cell survival was obtained from the following formula [29].

$${\mathbf{Cell}}~{\mathbf{survival}}~{\mathbf{rate}}\left( \% \right) = \frac{{\user2{OD~in~treated~cells} - \user2{OD~in~negative~control}}}{{\user2{OD~positive~Control} - \user2{OD~in~negative~control}}} \times 100$$

### Cultivation of *Plasmodium falciparum*

The cultivation of the *Plasmodium falciparum* parasite was conducted according to the methods of Tranger & Jensen [30], and Volkman, with some modifications, using the 3D7 strain at the National Malaria Laboratory, Faculty of Health, Tehran University of Medical Sciences. Observations of *Plasmodium falciparum* in red blood cells revealed a progression of developmental stages: young trophozoites were present from 0 to 14 h, growing trophozoites were observed from 14 to 28 h, schizonts from 28 to 40 h, and by 40 to 52 h, the highest number of parasites appeared in the young trophozoite stage (ring stage). The highest percentage of parasitemia was recorded at over 80% during the young ring stage.

### Preparation of incomplete culture medium (PCM)

For this purpose, soluble hypoxanthine and soluble gentamicin were prepared and added to the RPMI 1640 medium purchased from Gibco. Also, 5 mol NaOH was used for the pH adjustment of the medium to 7.2.

### Preparation of complete culture medium (CCM)

A complete culture medium (CCM) was obtained by adding human AB + serum decomplemented to incomplete culture medium (PCM). The amount of serum added each time is about 10% of the total volume of the medium.

### Parasite cultivation

Plates with a capacity of 4 ml or T25 culture flasks were employed for the cultivation of parasites. CCM culture medium and blood (to sustain a hematocrit of 10% within the culture medium for the proliferation and replication of the parasite) were supplemented into the dishes. Preparing slides every two days and centrifuging the parasite culture medium were done every two weeks. Parasites were multiplied in a CCM medium containing 10% hematocrit with O + human erythrocyte and albumax (0.5%) serving as a growth factor. The culture plates were incubated at 37 °c in a candle jar, with the fresh medium being replenished every 48 h. A thin blood smear was prepared from the sediment collected from each plate, followed by the quantification of parasites on 10,000 red blood cells. When parasitism reached more than 10%, PEGylated iron nanoparticles with plant extract, nicotinamide, and chloroquine drug were added to the environment as a controlled drug to measure their anti-parasitic effect.

### The effect of the drug on the culture medium and investigating the growth process of *Plasmodium falciparum*

To investigate the effect of drugs on the culture medium, two stages were conducted. The effect of the drug was performed when 80% of the parasites were in the ring stage and the parasitemia was 6%. The drug susceptibility test (IC50) was performed after 24 h by examining the results of the mean percentage of parasitemia by microscopic method and calculating the mean percentage of inhibition. In the first step, the drugs were treated separately and their IC50 was measured. For plant extract, dilutions of 100, 200, 400 and 800 µg/mL (dry extract) of culture medium were done in 12 wells of a 96-well plate (in triplicate).

For nicotinamide, dilutions of 10, 10, 375, 750, 1500 and 3000 µg/mL of culture medium were done in 12 wells of a 96-well plate (in triplicate). For chloroquine diphosphate as a control drug, dilutions of 1, 2, 4, and 8 µg/mL culture medium were done in 12 wells of a 96-well plate (in triplicate). For polyethylene glycol, dilutions of 10, 100, 1000 and 10000 µg/mL of culture medium were done in 12 wells of a 96-well plate (in triplicate). For the nanoparticle produced by the PEGylated green synthesis method (PEG-HNPs) dilutions of 50, 100, 200, and 400 µg/mL culture medium were done in 12 wells of 96-well plates (in triplicate). In the second step, the effect of the IC50 combination of drugs with each other was investigated by the Fix ratio method on the culture medium. In this way, the percentage ratio of 0, 10, 30, 50, 70, 90, and 100 of one drug was combined with another drug and it was given an effect on the culture medium, and their synergistic and antagonistic effect was investigated by affecting the amount of parasitemia. Negative control consisted of 15 μL of freshly washed RBC plus 85 μL of CCM. The positive control was 15 μL of infected RBC with 6% parasitemia in continuous culture and 85 μL of CCM. Statistical analysis was done using GraphPad prism9 software and the results were analyzed with one-way ANOVA. The significance level of the differences was P < 0.05.

## Results

### Characteristics of produced nanoparticles

#### Dynamic light scattering (DLS)

**Fig. 1:**
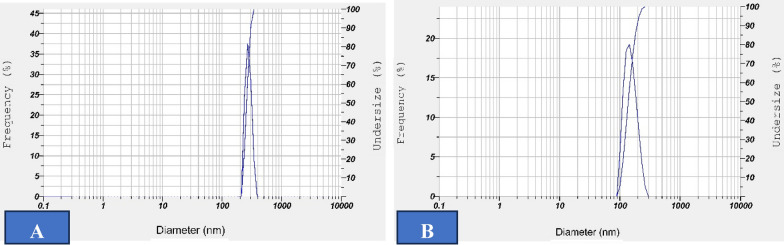
Average hydrodynamic size of different formulations obtained by dynamic light scattering (DLS) apparatus. **A **Fe_3_O_4_ obtained by green synthesis. **B** Fe_3_O_4_ obtained by PEGylated green synthesis

Hydrodynamic measurement of iron oxide nanoparticles produced by green synthesis and PEGylate green synthesis, was done with the DLS technique. The size distribution based on the frequency of nanoparticles is shown in Fig. 1 The average hydrodynamic sizes for Fe_3_O_4_ obtained by green synthesis and green PEGylate synthesis are 290 and 155 nm, respectively. In all samples, the particle size distribution curves presented only one peak with a relatively low polydispersity index (PDI), which indicates that nanoparticles do not show much aggregation in the solution. Negative zeta potential was obtained in both nanoparticles (Table 1).

**Table 1:** Particle size, zeta potential and polydispersity index (PDI) of iron oxide nanoparticles conducted using dynamic light scattering (DLS)

*Formulation*	Size(nm)	Zeta(mv)	PDI
Green synthesized NPs	290 ± 16	− 33.6 ± 2	0.711 ± 0.01
PEG-green synthesized NPs	155 ± 11	− 37.9 ± 3	0.669 ± 0.01

The mean ± SD is shown in the results (*n* = 3)

### Field emission scanning electron microscope (FESEM)

**Fig. 2:**
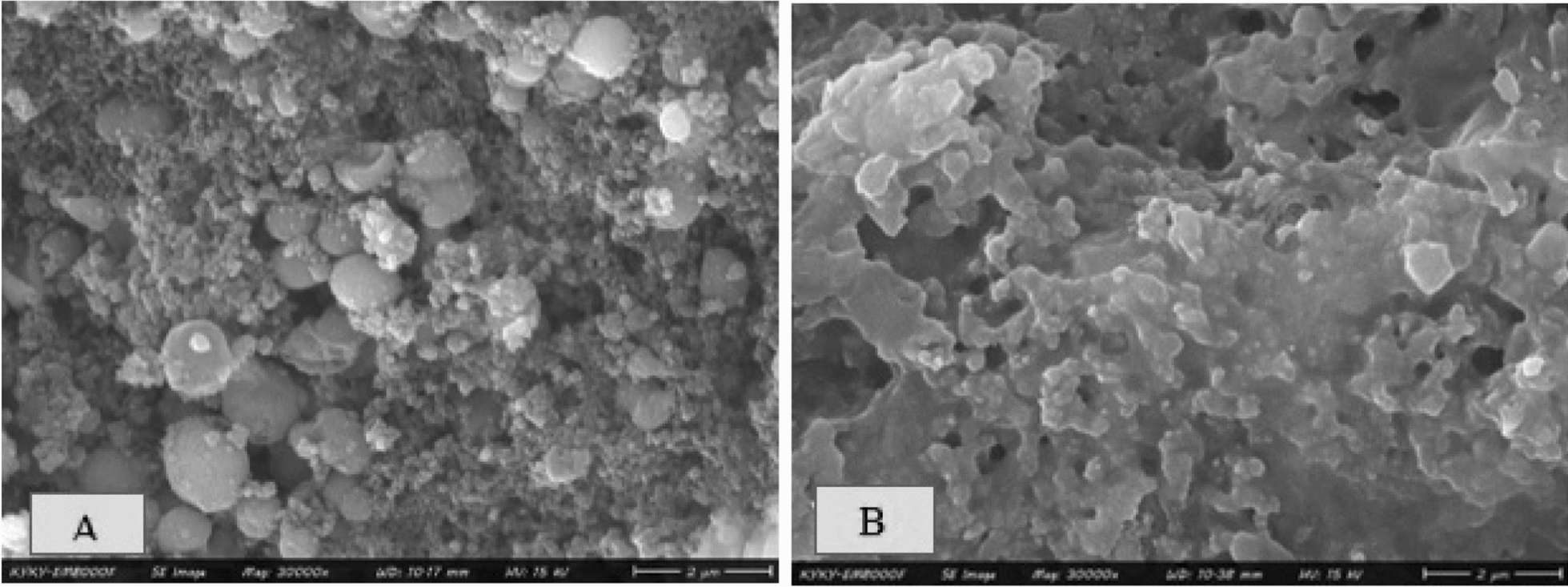
The micrographs of Fe_3_O_4_ nanoparticles by FESEM. **A** Iron nanoparticles obtained by green synthesis, **B** iron nanoparticles obtained by PEGylated green synthesis

The surface morphology and composition of chemical elements and consistency of iron oxide (Fe_3_O_4_) nanoparticles synthesized via the green PEGylated method were examined using a field emission scanning electron microscope (Fig. 2). It is seen that PEG is placed as a coating on the surface of spherical iron oxide nanoparticles, crystals, and plant clusters.

### Fourier transform infrared spectroscopy (FTIR)

**Fig. 3:**
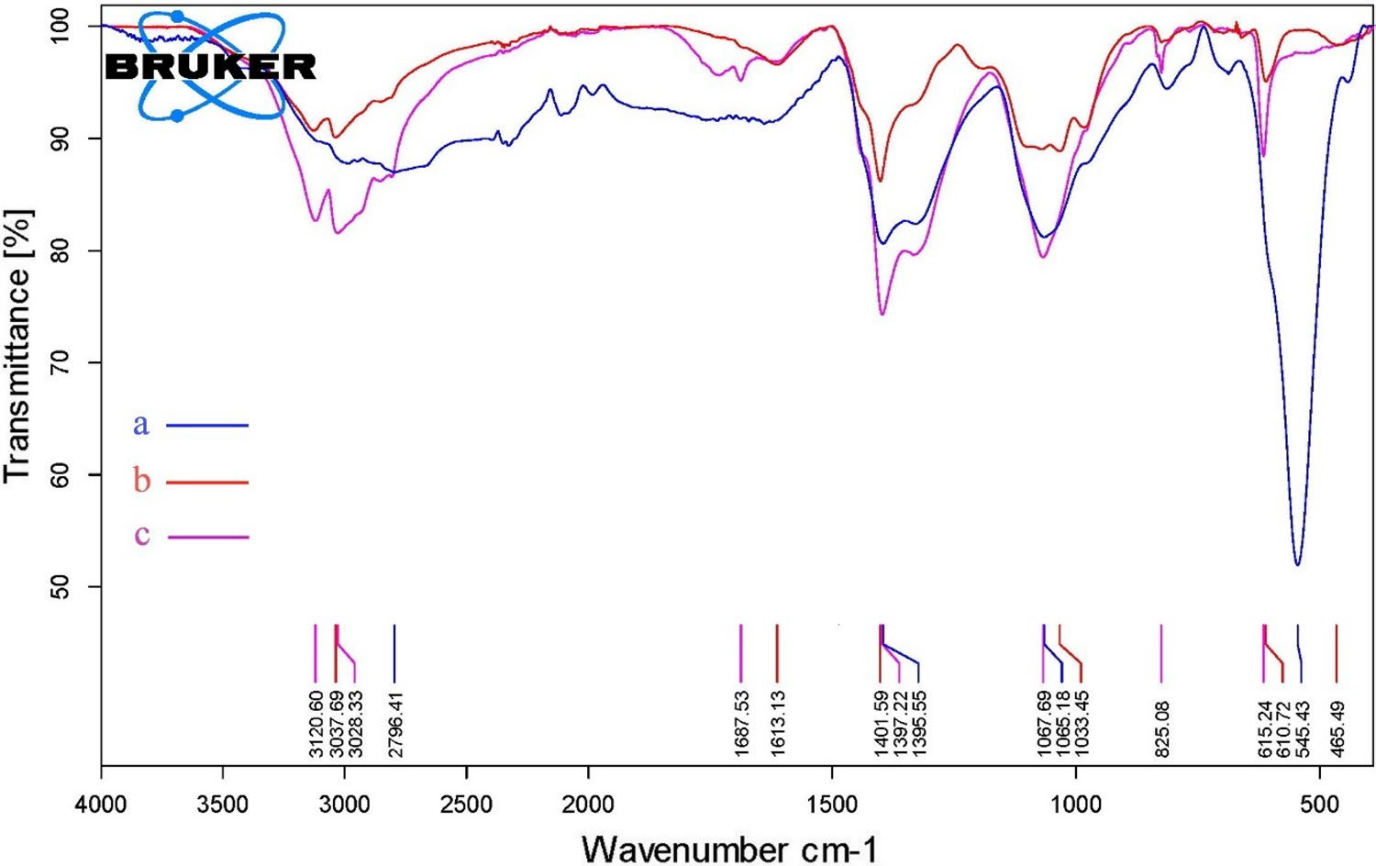
FTIR analysis: **a** bare Fe3 O4 nanoparticles, **b** iron nanoparticles obtained by green synthesis, **c:** iron nanoparticles obtained by PEGylated green synthesis

The results of FTIR spectroscopy were carried out to check the bonds and functional groups on the nanoparticles obtained by the green PEGylated synthesis method. It is specified in Fig. 3. In all spectra in the range of 540 to 620 waves/cm, a deep peak can be seen, which is a feature of Fe–O bond absorption. Also, two deep peaks in the area of ​​1033.45, 1067.69.18, as well as 1395.55, 1397.22, and 1401.59 waves/cm are seen in all three produced nanoparticles, which is stronger absorption in green-synthesized nanoparticles, especially PEGylated green synthesis. In this region, C–H and O–H bending bonds and S = O, C–N, and C–O stretching bonds are absorbed. In the region above 1613.13 and 1687.53 waves/cm, two peaks can be seen in the simple and PEGylated green synthesized iron oxide nanoparticle, which shows the absorption of C = O stretch bonds in these regions. Also, sharp peaks can be seen in all spectra in the region of 2796.41, 3028.33, 3037.69, and 3120.60 waves/cm, the first peak is the absorption characteristics of aldehyde C–H stretching bonds and the other three peaks are the absorption characteristics of C–H alkene stretching bonds. The peaks in the range of 2400 to 3400 waves/cm can be related to the characteristics of acidic O–H absorption.

### Cytotoxicity (MTT)

**Fig. 4:**
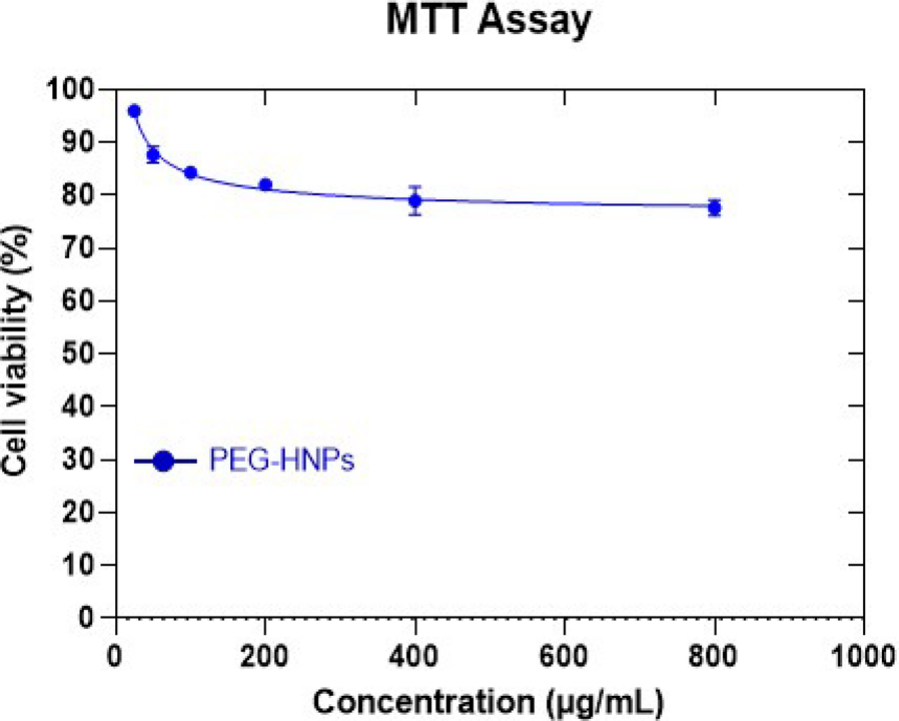
Cytotoxicity effect of PEGylated green synthesis Fe_3_O_4_ nanoparticle at doses of 25, 50, 100, 200, 400, and 800 μg/ml on MCF-7 cell line. Error bar represents mean ± SD (n = 3)

Cytotoxicity effect of hydroalcoholic extract of *E. japonica*, nicotinamide, and PEGylated green synthesis nanoparticle at doses of 25, 50, 100, 200, 400, and 800 μg/ml on MCF-7 cell line was investigated (Fig. 4), and their IC50 was calculated (Table 2). The results showed that the negative control in the used concentration (0.5%) has no cytotoxicity.

**Table 2:** IC50 of green synthesized Fe_3_O_4_ NPs, PEG-green synthesized NPs and nicotinamide (NAM) according to their effect on MCF-7 cells

*Medications*	Green synthesized NPs	PEG-green synthesized NPs	Nicotinamide (NAM)
IC_50_ (µg/ml)	1945	4433	1763

IC50 of hydroalcoholic extract of *Eriobotrya japonica* (herbal extract), nicotinamide (NAM), and synthesized PEGylated green iron oxide nanoparticles on MCF-7 cells were calculated as 1945, 1763, and 4433 µg/ml, respectively (Table 2). The results obtained from the effect of the extracts on MCF7 cells showed that the percentage of cell survival decreases with the increase in the concentration of the extracts. The lowest cell survival in the toxicity test was observed at the highest concentration of nanoparticles used (800 µg/ml).

### Hemolytic test

**Fig. 5:**
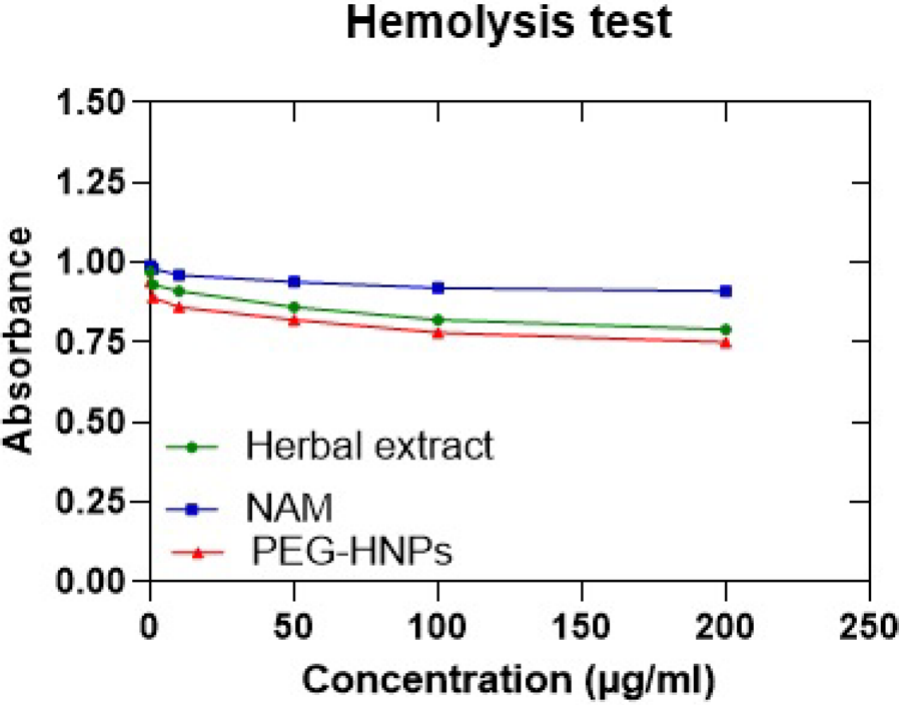
The optical absorbance *Eriobotrya japonica* hydroalcoholic extract (herbal extract), nicotinamide (NAM) and PEGylated green synthesized Fe_3_O_4_ nanoparticles (PEG-HNPs) on red blood cells. Error bar represents mean ± SD (n = 3)

Investigating the hemolytic effect of *Eriobotrya japonica* hydroalcoholic extract (herbal extract), nicotinamide (NAM), and PEGylated green synthesized iron oxide nanoparticles (HNPs) on red blood cells at concentrations of 0.1, 1, 10, 50, 100 and 200 µg/mL were evaluated (Table 3). The optical absorbance of PBS was measured as negative control 0.01 and the optical absorbance of Triton X-100 was measured as positive control 1.00 (Fig. 5).

**Table 3:** The hemolytic effect of *Eriobotrya japonica* hydroalcoholic extract (herbal extract), nicotinamide (NAM) and PEGylated green synthesized Fe_3_O_4_ nanoparticles (PEG-HNPs) on red blood cells

Drug concentration(µg/mL) Percentage of drug hemolysis	0	0.1	1	10	50	100	200
Herbal extract	0	3	7	9	14	18	21
PEG-HNPs	0	6	11	14	18	22	25
NAM	0	1	2	4	6	8	9

### Estimation of total phenolic content of plant extract (TPC)

**Fig. 6:**
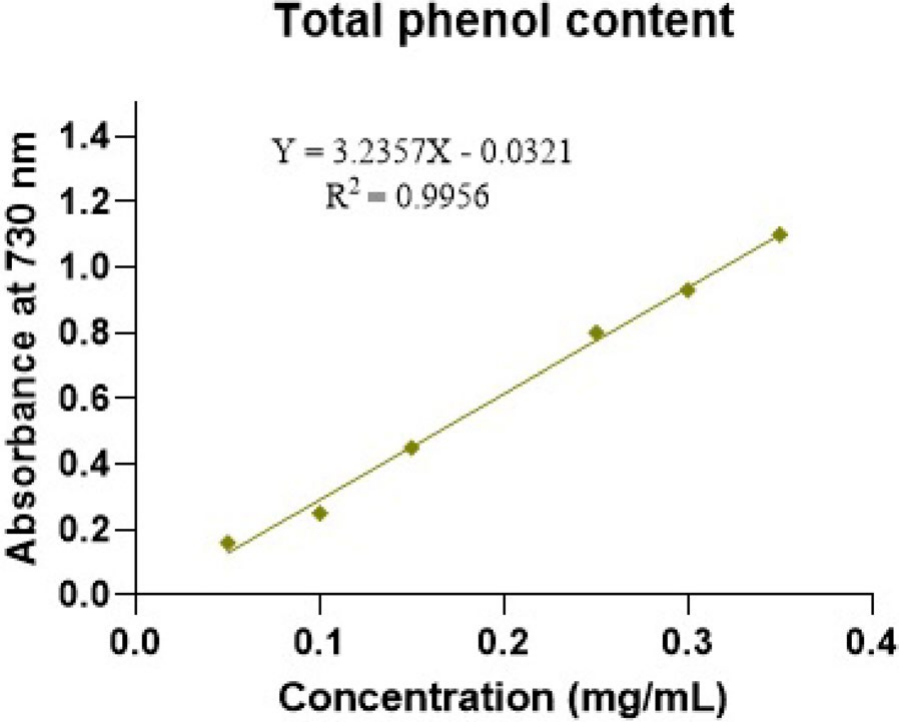
The standard curve of gallic acid to determine of total phenol of *Eriobotrya japonica* by the Folin–Ciocalteu method

The total phenolic content was calculated by the Folin–Ciocalteu method as mg of gallic acid equivalent per gram of extract based on the standard curve equation (Y = 3.2357X—0.0321; R2 = 0.9956). For this purpose, ethanolic concentrations of 0, 50, 100, 150, 200, 250, and 350 ppm of powdered extract were prepared and after adding Folin–Ciocalteu reagent and Na2 Co3 (20%) at 760 nm wavelength, their optical absorption was measured. The total phenolic content for the hydroalcoholic extract of Japanese parsnip leaves was 142.2 mg gallic acid equivalent per gram of extract (Fig. 6).

### Determination of antiradical activity

**Fig. 7:**
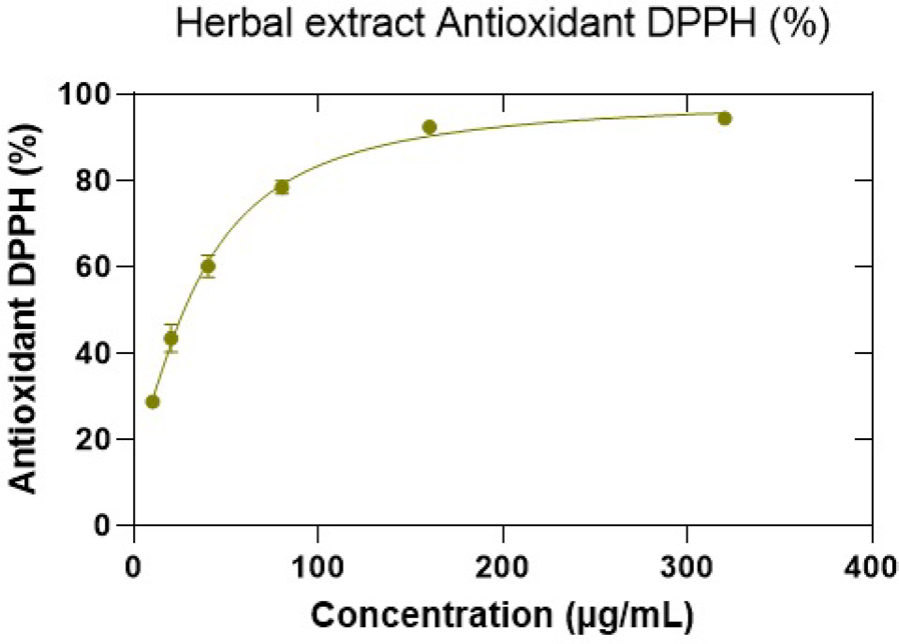
Percentage of DPPH free radical scavenging by different concentrations of *Eriobotrya japonica*. Error bar represents mean ± SD (n = 3)

To measure the antiradical activity of plant leaf extract from Japanese gill, the method of comparing the optical absorption of different concentrations of methanolic solution of powdered plant extract to 2,2-Diphenyl-1-picrylhydrazyl (DPPH) was used. For this purpose, concentrations of 10, 20, 40, 80, 160, and 320 ppm of plant extract were prepared in methanol, and compared to DPPH as a blank and methanol as a control, their optical absorption was measured at a wavelength of 517 nm. Figure 7 of the DPPH antiradical percentage is drawn. The results of this research showed that the methanolic extract of Japanese parsnip leaves has a good ability to trap and inhibit DPPH free radicals. The IC50 value for the methanolic extract of Japanese plant leaves was found to be 26.79 µg/mL. The percentage of antiradical activity at the lowest concentration of 10 µg/mL was 28% and reached 94% with increasing concentration to 320 µg/mL.

### The effect of treatments on the cultivation environment

#### Herbal extract

The effect of hydroalcoholic extract of Japanese parsnip (*Eriobotrya japonica*) in RPMI 1640 culture medium at concentrations of 100, 200, 400, and 800 µg/ml was investigated. The results of the effect of this extract on the growth of *Plasmodium falciparum* 3D7 in a culture medium show that the highest growth inhibition 70% was observed at the concentration 400 µg/ml (Table 4).

**Table 4:** Growth inhibition rate *Eriobotrya japonica* hydroalcoholic extract (herbal extract), nicotinamide (NAM), chloroquine diphosphate (CQ), polyethylene glycol 600 (PEG**)** and PEGylated green synthesized Fe_3_O_4_ nanoparticles (PEG-HNPs) in RPMI 1640 culture medium on *Plasmodium falciparum* 3D7

Formulation	Concentration(µg/ml)	Growth inhibition rate
Herbal extract	400	70
NAM	3000	94
CQ	8	95
PEG	10000	71
PEG-HNPs	1/53	80

Error bar represents mean ± SD (n = 3)

### Nicotinamide (NAM)

The effect of isolated nicotinamide in the culture medium on *Plasmodium falciparum* and the initial stage was investigated with concentrations of 10, 100, 375, 750, 1500, and 3000 μg/ml. The results of the effect of NAM on the growth of *Plasmodium falciparum* 3D7 in a culture medium show that the highest growth inhibition 94% was observed at the concentration 3000µg/ml (Table 4).

### Chloroquine diphosphate (CQ)

The effect of chloroquine on *Plasmodium falciparum* 3D7 (susceptible to chloroquine) was investigated as a control drug in RPMI 1640 culture medium at concentrations of 1, 2, 4, and 8 µg/ml. The results of the effect of CQ on the growth of *Plasmodium falciparum* 3D7 in a culture medium show that the highest growth inhibition 95% was observed at the concentration 8µg/ml (Table 4).

### Polyethylene glycol 600 (PEG)

The effect of separate polyethylene glycol 600 in the culture medium on *Plasmodium falciparum* and the initial stage was investigated with concentrations of 10, 100, 1000, and 10,000 µg/ml. It is reported in tables and graphs. The results of the effect of PEG on the growth of *Plasmodium falciparum* 3D7 in a culture medium show that the highest growth inhibition 71% was observed at the concentration 10000 µg/ml (Table 4).

### Iron oxide nanoparticles obtained by PEGylated green synthesis method (PEG-HNPs)

The effect of iron oxide nanoparticles obtained by the PEGylated green synthesis method was investigated in RPMI 1640 culture medium at concentrations of 50, 100, 200, and 400 µg/ml. The results of the effect of PEG-HNPs on the growth of *Plasmodium falciparum* 3D7 in a culture medium show that the highest growth inhibition 80% was observed at the concentration 1/53µg/ml (Table 4).

### The effect of relative combined doses of drugs (Fix ratio)

#### Plant extract–nicotinamide

**Fig. 8:**
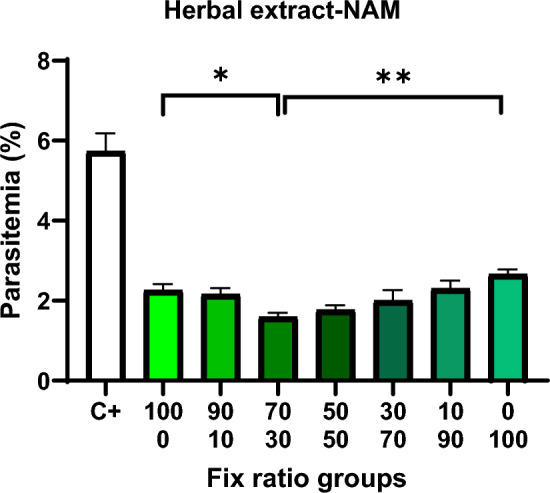
Growth inhibition rate of relative plant extract–nicotinamide combination in RPMI 1640 culture medium on *Plasmodium falciparum* 3D7. *P < 0.05, **P < 0.001

In the second stage of the effect of the drugs on the culture medium, the IC50 obtained in the first stage of the effect of the drugs for each drug was combined one by the Fix ratio method to find the best therapeutic combination to inhibit the growth of *Plasmodium falciparum* 3D7 in the RPMI 1640 culture medium. Seven drug combinations were created, each with a specific percentage of the drugs included 0–100%, 10–90%, 30–70%, 50–50%, 70–30%, 90–10% and 100–0%, respectively, prepared, and the effect was given on the culture medium. The results of the effect of the relative composition of herbal extract–nicotinamide are mentioned in Fig. 8. The relative combination of these two drugs had a synergistic effect and the best dose was observed in 70% plant extract-30% nicotinamide.

### Iron nanoparticles obtained by the method of PEGylated green synthesis-nicotinamide

**Fig. 9:**
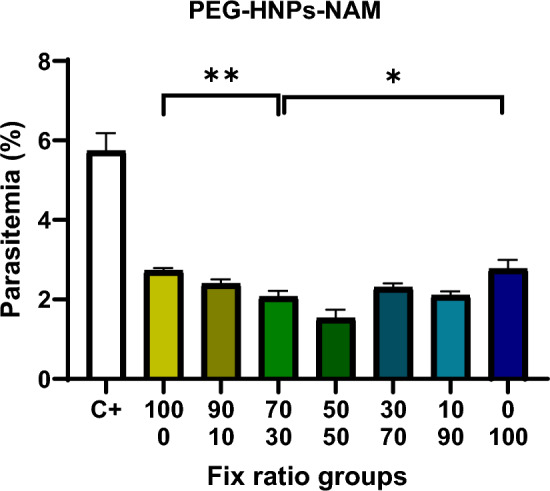
Growth inhibition rate of relative PEGylated green synthesized Fe_3_O_4_ nanoparticles (PEG-HNPs)-nicotinamide combination in RPMI 1640 culture medium on *Plasmodium falciparum* 3D7. *P < 0.05, **P < 0.001

The results of the effect of the relative composition of the iron oxide nanoparticle obtained by the PEGylated green synthesis method with nicotinamide, which is the desired composition of the project, are shown in Fig. 9. The relative combination of these two drugs has a completely synergistic effect, and the best combination was found to be 50% iron nanoparticles of PEGylated with nicotinamide 50%.

## Discussion

Plasmodium parasite, the causative agent of malaria, has a very wide impact on the health of human societies worldwide. Due to the speed of progress and the wide geographical distribution of parasite resistance to common antimalarial drugs, the preparation of different new drugs against this disease is a definite necessity. The emergence of drug resistance means an increase in the risk of side effects or more expenses in the use of new drugs [31].

Iron oxide nanoparticles (Fe3O4) were selected as the drug carrier and plant extract stabilizer in this study. The method of producing these nanoparticles with the help of PEGylated green synthesis has advantages such as smaller size and accumulation, electric charge, and more stability among these advantages. Uchechukwu et al., also reached similar results using data obtained from the dynamic light scattering (DLS) device. However, its magnetic properties, low stability, and toxicity in high doses make its use in in vitro studies difficult [32, 33].

PEGylation reduces nanoparticles (NPs) surface interaction with its environment, hence minimizing its detection by the immune system. PEG is widely used in providing NPs with stealth properties, hence prolonging blood circulation times. Various studies show that PEG-coated NPs accumulate in immune system organs responsible for NP body clearance for more than six months. With PEGylation, NPs may evade the immune system, achieving longer blood circulation times. The effects of iron oxide nanoparticles on cellular environments and the role and sensitivity of iron released from molecular decomposition are very important, as mentioned in the study of Soenen et al. and Yang et al. [33, 34]. PEGylation of nanoparticles is a suitable and non-toxic method to create stable magnetic nanoparticles and reduces their size, which was also confirmed by Arslani et al. [35]. Similar to the research results obtained in the study of Pirro et al., it was shown that the green synthesis method produces iron oxide nanoparticles with smaller size, dispersion, and stability than the chemical method [36].

As reviewed by Yew et al., plant extract materials have played a role as reducers and stabilizers in the formation of metallic iron oxide nanoparticles [37]. Also, in the present study, these advantages exist in the use of PEG in the green synthesis of iron oxide nanoparticles. Joshi et al. clearly showed that the green pegylated synthesis method for producing iron oxide nanoparticles (Fe3O4) leads to obtaining nanoparticles with small size, high stability, and less toxicity than other methods [38]. These findings are in line with previous findings such as the study of Antarnusa et al. [39]. The decrease in the diameter of Fe3O4 obtained by the green PEGylated synthesis method is probably due to the surface charge of plant compounds and PEG-600 molecules on the surface of Fe3O4 nanoparticles in the green PEGylated synthesis and the increase in the potential difference between the main fluid and the fluid layer corresponding to the opposite charge on the nanoparticle surface [40], the results of this research showed that the green synthesis method with the help of polyethylene glycol (PEG) is an effective, low-cost, and non-toxic way to produce iron oxide nanoparticles (Fe3O4). In the images obtained from FESEM, the network of spherical iron oxide nanoparticles, crystals, and plant extract compounds were visible. Also, the polymer coating of polyethylene glycol was evident in the sample obtained by the PEGylated green synthesis method. Small building blocks of bioactive reducing agents and polyethylene glycol in iron oxide nanoparticles obtained by simple green and PEGylate synthesis methods are placed between magnetic nanoparticles and probably by increasing the zeta potential of nanoparticles, it has reduced their agglomeration, which is in line with the findings of Bhuiyan et al. It is straight [41].

In the results of X-ray energy diffraction spectroscopy (EDS) analysis, the presence of carbon can be related to the functional groups of plant compounds and polyethylene glycol, which is much more observed in the final nanoparticles of PEGylated synthesis. Similar results have been observed in the study of Kannan et al. and Santos et al. [16, 42]. The presence of a deep peak in the absorption region of the Fe–O bond of the iron oxide nanoparticle obtained by the green PEGylated synthesis method in the FTIR analysis results confirms the presence of the magnetic core. These findings align with the observations made in the study conducted by Silva et al. The presence of deep peaks in the absorption region related to S = O stretch bonds in three nanoparticles can probably be due to the use of iron sulfate salt in their synthesis [15]. Also, similar to the results of Qasim et al., the much greater depth of the peak in the absorption region related to C–H and O–H bending and stretching bonds, and S = O and C–N stretching bonds can be obtained by properly loading the plant extract with aldehyde, alkene, alcohol, and carboxylic acid functional groups. In the network of iron oxide nanoparticles, especially the iron oxide nanoparticles obtained by the green synthesis of PEGylated method [43]. The presence of absorptions related to C = O stretching bonds in the iron oxide nanoparticles of green synthesis of PEGylated method can be related to the presence of plant extract, as in the study of Aisida et al. [44, 45]. The results of the cytotoxicity test of Japanese parsnip leaf extract and nicotinamide on the MCF-7 cell line showed that in the doses used in the usual in vitro and in vivo uses, the cytotoxicity is very small, which is in line with the results of the findings of Fard et al. It is consistent with Huang et al. [46, 47]. The toxicity of iron oxide nanoparticles obtained by the green PEGylated synthesis method was higher than in the other two cases. Still, the low doses used in the study showed little toxicity. which is consistent with the results of Feng et al. [48].

The hemolytic test in this research showed that the hydro alcoholic extract of Japanese parsnip leaves, nicotinamide, and iron oxide nanoparticles of PEGylated green synthesis caused the lysis of red blood cells in a small amount in the doses used and did not cause an interfering effect in the culture medium. Fouedjou et al. attributed the antihemolytic activity of Japanese parsnip leaf extract to its phenolic compounds [49]. Agarwal et al. introduced amphiphilic magnetic nanoparticles with antihemolytic activity [50]. Estimation of the total phenolic content of Japanese parsnip leaf extract confirmed the high number of phenolic compounds in the plant. These results are not consistent with the statements of Liu et al. The difference in the method of extraction can be attributed to this issue. In this regard, the antiradical activity of the plant extract by the DPPH method showed a lower-than-expected IC50 in the research compared to previous studies including Liu et al., which can be attributed to the phenolic content. He considered high flavonoids as the cause [51].

Considering the efficacy of drugs available for the treatment of infectious diseases, as well as their side effects and the resistance developed by parasites, research in phytosciences, mainly regarding the properties of bioactive phytocompounds found in the crude extracts of medicinal plants, may lead to the discovery of new medicines that are effective, inexpensive, and safe for patients [52]. One of the most significant types of inorganic nanoparticles is iron oxide nanoparticles (IONPs), particularly superparamagnetic Fe_3_O_4_ nanoparticles (magnetite core). Due to their superparamagnetic characteristics and potential biomedical uses resulting from their non-toxicity and biocompatibility, these nanoparticles have garnered considerable interest. Ferumoxytol, an FDA-approved carbohydrate-coated iron oxide nanoparticle, has recently been used extensively as an iron supplement to treat several disorders, including cancer, iron deficiency anemia (IDA), and chronic kidney disease [53]. In the case of malaria, the ability to effectively attach biomolecules to the surfaces of magnetic particles is essential for their practical use. Al-Abboodi et al. explored coupling the recombinant 19-kDa C-terminal segment of merozoite surface protein 1 (PyMSP119) of *P. yoelii* to the surface of superparamagnetic magnetite nanoparticles (SPIONs) as a novel method to enhance malaria vaccination [54].

As Gudkov et al. have shown, iron oxide nanoparticles possess good antimicrobial and antiparasitic properties as carriers and effective drugs; however, their magnetic properties, low stability, and toxicity at high doses complicate their use in in vitro studies [55]. PEGylation of nanoparticles is a suitable and non-toxic method for creating stable magnetic nanoparticles, which reduces the incidence of these problems and diminishes their size, as confirmed by Arslani et al. [56]. Abakumov et al. demonstrated that the use of PEG in the synthesis of iron nanoparticles reduces their cytotoxicity and increases their stability, which is also observed in the present study [57]. As indicated by the results of the present study and noted by Santos et al. and Huang et al. the use of PEG, in addition to coating drug delivery systems in many contexts and in nanoparticles, is biocompatible and has a toxicity-reducing effect by enhancing drug stability. The FDA has also approved its use for human purposes [58, 59].

Similar to this study, Tcherniuk et al. demonstrated the growth-inhibitory effect of nicotinamide in combination with artemisinin, chloroquine and pyrimethamine on the growth of blood stages of *P. falciparum*. Nicotinamide prevents the proper formation of the ring form of the parasite and renders it vulnerable by inhibiting the DNA repair enzyme (PRRP), ADP-ribose transferase (ART), and cyclic ADP-ribose hydrolase (CD38). Nicotinamide acts below the level of tolerance and reduces the effective concentration of antimalarial drugs due to synergism. These in vitro results suggest that nicotinamide might be useful not only as a vitamin supplement, but also as an enhancer of the anti-parasitic effect of common antimalarial drugs including artemisinin, chloroquine and pyrimethamine [24].

Iron oxide nanoparticles are used as carriers for antimalarial drugs and, as discussed in the study by Kannan et al. show an additive effect on drug efficacy [16]. In the present study, iron oxide nanoparticles prepared by the PEGylated green synthesis method exhibited the highest inhibition of the drugs tested. These nanoparticles demonstrated excellent efficacy in culture media against the growth of *Plasmodium falciparum* 3D7, with an IC50 of 82.27 μg/ml and suitable characteristics, such as small size, stability, and homogeneity. In a related study, Dutta et al. investigated the effect of gold and silver nanoparticles obtained through the green synthesis method using *Syzygium jambos* plant extract on chloroquine-sensitive strains (3D7) during the schizont stage. They also observed that the green synthesis of AgNPs nanoparticles had a greater than AuNPs inhibitory effect on *Plasmodium falciparum* in culture medium [60].

In this study, the investigation of the inhibitory effects of the IC50 s obtained in combination (Fix ratio) on the growth of *Plasmodium falciparum* in culture medium was promising and indicated that the combination of plant extract–nicotinamide and iron oxide nanoparticles at all combination percentages (Fix ratio) exhibits an additive effect on the growth of *Plasmodium falciparum* 3D7. The highest synergistic effect was obtained in the combination ratio of 50% iron nanoparticles (synthesized through the PEGylated green synthesis method) and 50% nicotinamide. In studies of combination antimalarial therapy, researchers such as Alven et al. and Maiga et al. have shown that combination methods possess greater therapeutic potential than other treatments [61, 62].

## Conclusion

The results obtained from the inhibitory effect of the hydroalcoholic extract of *Eriobotrya japonica*, iron nanoparticles produced by the green synthesis method of PEGylated and nicotinamide on the growth of *Plasmodium falciparum* 3D7 in RPMI-1640 culture medium are quite evident. Also, the synergistic effect was observed in all combined percentages of plant extract–nicotinamide and iron oxide nanoparticles. On the other hand, green synthesis and pegylation of iron oxide nanoparticles are suitable methods for increasing stability, reducing toxicity, reducing size, and achieving greater ease and accuracy in the use of these particles in biomedical studies. A more detailed investigation of the mechanism of these methods and the biomedical potential, carrier and cellular targeting of these nanoparticles in the treatment of cancer and other diseases requires further research.

## Data Availability

No datasets were generated or analysed during the current study.
